# The Role of Dysphagia on Head and Neck Cancer Patients’ Quality of Life, Functional Disabilities and Psychological Distress: Outcomes of Cancer Rehabilitation from an Observational Single-Center Study

**DOI:** 10.3390/curroncol32040220

**Published:** 2025-04-10

**Authors:** Špela Matko, Christina Knauseder, David Riedl, Vincent Grote, Michael J. Fischer, Samuel Moritz Vorbach, Karin Pfaller-Frank, Wilhelm Frank, Thomas Licht

**Affiliations:** 1 Ludwig Boltzmann Institute for Rehabilitation Research, 1140 Vienna, Austria; 2 Oncological Rehabilitation Center, 5621 Sankt Veit i. Pongau, Austria; 3 Department of Psychiatry, Psychotherapy Psychosomatics and Medical Psychology, University Hospital of Psychiatry II, Medical University of Innsbruck, 6020 Innsbruck, Austria; 4 Department of Orthopaedics and Trauma, Medical University of Graz, 8036 Graz, Austria; 5 Rehabilitation Center Kitzbuehel, 6370 Kitzbuehel, Austria; 6 Department of Radiation Oncology, Medical University of Innsbruck, 6020 Innsbruck, Austria; 7 Faculty of Health and Medicine, University for Continuing Education Krems, 3500 Krems, Austria

**Keywords:** patient-reported outcomes, speech therapy, psychooncology, fatigue, depression, cancer survivorship

## Abstract

Many patients with head-and-neck cancer (HNC) suffer from speech or swallowing disorders. We investigated the impact of dysphagia on health-related quality of life (HRQOL), functioning, and distress in HNC survivors, and whether cancer rehabilitation can alleviate these conditions. Before admission (T0) and at discharge (T1) of three-week inpatient cancer rehabilitation, patient-reported outcomes were collected. HRQOL, symptoms, functioning, and psychological distress were assessed with EORTC QLQ-C30 and Hospital Anxiety and Depression Scale (HADS) questionnaires. Of 63 HNC patients, 22 had dysphagia, 23 needed no speech therapy (Control-1), and 18 needed speech therapy, but showed no symptoms of dysphagia (Control-2). Before rehabilitation, HRQOL, physical, social, and emotional functioning were significantly lower in dysphagia patients than in controls. Dysphagia patients reported more severe general symptoms including fatigue, pain, sleep disturbances, nausea/vomiting, diarrhea, and financial worries. Furthermore, the emotional and social functioning of Control-2 was significantly worse than Control-1. For all HNC patients, social, emotional, and role functioning, fatigue, nausea/vomiting, insomnia, and appetite loss significantly improved at T1. Improvements in HRQOL were most noticeable in dysphagia patients. Psychooncological counseling reduced depression in dysphagia and Control-2 patients to levels seen in the general population. In conclusion, dysphagia patients suffer severely from impaired functioning and systemic symptoms but benefit substantially from rehabilitation.

## 1. Introduction

The incidence of newly diagnosed cancers in the head and neck region, mostly squamous cell carcinomas, ranks seventh worldwide, with a notable increase in incidence and mortality rates [1]. Between 2020 and 2022, Austria recorded 1378 new diagnoses in the head and neck region (ICD-10 codes: C00–C14), with a mortality rate of 559.7 [2]. Additionally, 334 newly diagnosed cancers were reported in the larynx region (C32) with a mortality rate of 134.3 [2]. The incidence is three to five times higher in men than in women, depending on the tumor location [3]. The primary causes of HNC are chronic abuse of alcohol and nicotine, along with infection by human papillomavirus, particularly types 16, 18, and 31 [4,5].

Symptoms of HNC vary depending on the etiology and tumor location, with initial presentations often including lumps in the neck, open and painful areas in the mouth and throat, plaques, swelling, difficulty swallowing, and changes or hoarseness in voice and speech [6,7].

Treatment of HNC is multimodal and typically involves surgery, radio(chemo)therapy, or systemic therapy [8]. However, these treatments are frequently associated with long-term adverse effects that can significantly impact patients’ quality of life. Many HNC survivors experience impaired health-related quality of life (HRQOL) and reduced oral function [9]. Additional treatment-related side effects include skin changes, edema, pain in the head, neck, or shoulder girdle, as well as loss of taste, saliva, and appetite. Complications such as thrush, mucositis, xerostomia, restricted movement, tissue induration, fibrosis, necrosis, and trismus can severely impair voice quality and swallowing ability. Dysphagia and aspiration—often underestimated—can emerge even years after treatment, making them particularly challenging to manage [10,11,12,13,14]. Approximately three-quarters of HNC patients experience long-term morbidity and report at least two treatment-related side effects five or more years after diagnosis [9]. Risk factors for poor HRQOL include female patients, low income, laryngeal cancer, and advanced tumor stage [8].

Beyond the physical symptoms, HNC patients also endure significant psychological consequences, including a fear of recurrence, depression, and difficulties affecting their family, social environment, and return to work [3,9,10,11,12,13]. In particular, dysphagia can lead to nutritional deficiencies, weight loss, or dependency on feeding tubes, increasing the risk of life-threatening aspiration [14,15]. These extensive physical and psychological burdens underscore the need for comprehensive, patient-centered rehabilitation strategies. To address these challenges, participation-oriented speech therapy and individualized goals for swallowing and oral functions are essential for promoting reintegration into daily life [10,16]. The overarching objective of cancer rehabilitation is to enhance HRQOL and participation, both of which are compromised by functional limitations and somatic side effects. In addition, rehabilitation should aim to alleviate psychological distress or depression that may arise from the disease trajectory and the persistent fear of progression or relapse [17,18].

Patient-reported outcomes (PROs) are most helpful in identifying patients’ specific unmet needs. They provide insights into patients’ perceptions of their complaints from their own perspective and the associated limitations in HRQOL. Furthermore, they can improve communication between healthcare professionals and patients [19]. Several studies have shown discrepancies between the clinical assessment of symptoms of healthcare professionals and the self-assessment of patients [20,21].

PROs have been utilized for the assessment of HRQOL and physical, psychological, and social functions [22]. They can be instrumental in the planning and compilation of therapies in rehabilitation measures [23,24,25]. Thereby specific objectives are devised and incorporated into clinical processes [22]. PROs also enable the assessment of individual rehabilitation success [22,26]. In addition, the needs of specific subpopulations of cancer survivors can be better understood by collecting electronic PROs before and after rehabilitation [27]. Using electronic PROs, we have previously investigated the effectiveness of oncological rehabilitation in different tumor entities, age groups, and male versus female patients in longitudinal studies [27,28]. For the present study, HRQOL was assessed using the European Organization for Research and Treatment of Cancer (EORTC) standard questionnaire, QLQ-C30 [29,30]. The level of psychological distress was measured using the Hospital Anxiety and Depression Scale (HADS) [31,32]. Both instruments are validated and widely used for the assessment of the somatic and psychosocial burden of cancer.

Improvements in HRQOL among HNC patients through rehabilitation interventions in various settings have been explored in several studies [33,34,35]. These studies have primarily focused on alleviating dysphagia symptoms and, in some cases, on associated disabilities and HRQOL. However, direct comparisons of systemic impairments specifically between dysphagia patients and other HNC survivors have not been sufficiently investigated. In the present study, we focused on general functional impairments, reductions in HRQOL, systemic symptoms, and psychological distress associated with swallowing disorders. To this end, we compared HNC survivors with dysphagia to those who experienced local symptoms (requiring speech therapy) but had no signs of dysphagia, as well as to HNC survivors without any speech or swallowing impairments. The aim of the current study was to (1) investigate the specific impact of swallowing disorders on HRQOL and functions, (2) psychological distress in HNC survivors, and (3) to evaluate the benefits of cancer rehabilitation for HNC survivors with dysphagia in comparison to those who were not impaired by swallowing disorders.

## 2. Materials and Methods

Data were collected at the Austrian Rehabilitation Centre St. Veit im Pongau during a 21-day cancer rehabilitation program. In cases of severe impairment, patients could extend their stay for seven days. The treatment costs were covered by Austrian pension funds, which stipulated the respective therapy frequencies and the minimum total treatment time (1800 min). According to pension fund guidelines, patients needed to have completed active oncological treatment by surgery, radiotherapy, or intravenous chemotherapy, and to be in a condition suitable for active participation in therapeutic interventions.

### 2.1. Patient Sample

All patients admitted to the rehabilitation center between 1 January 2019 and 31 August 2021 were screened. Inclusion criteria comprised ICD-10 tumor diagnoses, C00–C14, C30–C32, or C76. To avoid the risk of bias, only the first rehabilitation stay per patient was included in the case of multiple rehabilitations. Exclusion criteria comprised an early discharge (i.e., ≤three days), an interval of >56 days between the first assessment and treatment initiation, and incomplete data sets. A single speech therapist conducted a clinical swallowing function examination on all HNC patients, employing a standardized manual approach. This involved a thorough medical history review and clinical assessment of structures involved in swallowing, utilizing tests with foods of various consistencies and bolus sizes [36]. Based on the clinical swallowing function examination and logopedic anamnesis results, patients were classified into three predefined groups:

- **Dysphagia patients:** patients who needed speech therapy to treat swallowing disorders affecting ingestion, preparation, or transport of food;
- **Control group 1:** patients in whom no abnormalities were found regarding swallowing function or other symptoms requiring treatment by speech therapists;
- **Control Group 2:** patients without dysphagia symptoms who needed speech therapy due to other impairments such as xerostomia, dysgeusia, dysphonia, dysglossia, dyspnoea, painful hardening of throat and muscles.

### 2.2. Data Collection

Data were collected prior to treatment initiation through a web-based patient portal utilizing the Computer-based Health Evaluation System (CHES) [37], allowing patients to complete questionnaires from their homes. We explained that these questionnaires would help pre-plan treatments to meet their specific needs. In cases where patients had not completed the questionnaire a week before admission, a member of the rehabilitation center’s administration reached out to remind them and offer guidance on technical issues. After admission to rehabilitation, patients were informed about the nature of the study and were asked to provide written informed consent for the scientific use of their data. If patients refused, their data were used only for routine care and were not included in this study. A second assessment was conducted upon discharge (T1), employing identical questionnaires.

### 2.3. Ethics Approval

This study was reviewed by the Ethics Committee of the State of Salzburg (vote no. 415-EP/73/706-2017, from 18 January 2017) which waived the necessity of approval because the study is merely observational and non-interventional. The study was conducted according to the principles of the Declaration of Helsinki.

### 2.4. Outcome Assessment

The assessment of functions and symptoms was conducted using the German version of the EORTC QLQ-C30 questionnaire [29], which consists of 30 questions that are organized into scales for global HRQOL, five functioning aspects, namely physical, social, role, emotional, and cognitive functioning; and nine symptom scales, i.e., fatigue, nausea/vomiting, pain, dyspnoea, sleep disturbances, appetite loss, constipation, diarrhea, and financial impact as well as a global HRQOL scale. The scoring adhered to the EORTC manual guidelines, which transformed raw scores to a scale of 0 to 100, where 100 represented the worst symptom score and the best functioning score, respectively [38].

The Hospital Anxiety and Depression Scale (HADS) is a 14-item questionnaire that generates a total score ranging from 0 to 42, with sub-scores for anxiety and depression. Clinical cases of anxiety or depression are defined as scores of 11 or higher, while scores ranging from 7 to less than 11 are considered doubtful [32].

### 2.5. Rehabilitation Therapeutic Program

A tailored therapy approach was provided based on the admission assessments. Patients underwent comprehensive multidisciplinary therapies as part of their rehabilitation. They were guided and treated by physicians, nursing staff, physiotherapists, psychologists, and specialists in various fields including speech therapists. The treatment regimen included aerobic and resistance training, psychological therapy, biofeedback or relaxation exercises, nutritional advice, social counseling, and educational presentations, encompassing motivation for lifestyle modifications. Additionally, patients commonly received occupational therapy, remedial massages, thermotherapy, hydro gymnastics, electrotherapy, and counseling for smoking cessation.

Speech therapy was offered to all patients with dysphagia or other oral dysfunctions. Techniques such as functional dysphagia therapy and manual swallowing therapy were used to enhance the range of motion and strengthen the muscles essential for swallowing, including those of the lips, tongue, and larynx. Additionally, compensatory maneuvers like the super-supraglottic swallow and the Mendelsohn maneuver were tailored to patients, considering structural changes post-surgery and/or radiotherapy. These therapies were complemented by posture, movement, and relaxation exercises, as well as counseling for issues like dry mouth and adaptive strategies. The oncological rehabilitation program was, however, provided for patients of all cancer entities and not specifically designed for HNC. Further extensive instrumental diagnostic examination procedures like video-endoscopy or structural dysphagia therapy programs were thus not incorporated. The Austrian pension funds’ guidelines, specifying therapy frequencies, served as the foundation for treatment planning, with the minimum total treatment time of 1800 min over 21 days.

### 2.6. Statistical Analyses

Mean differences between included and excluded patients were compared using *χ*^2^-tests and independent sample *t*-tests. Baseline differences (T0) between groups were analyzed using univariate analysis of variance (ANOVA). To evaluate changes in symptoms during rehabilitation, as measured by EORTC QLQ-C30 and HADS, repeated measures ANOVA was used. Mean values for EORTC QLQ-C30 and HADS at T1 were compared with published reference values from the general population of the country [32,39]. The effect size was determined using *η*^2^, with values categorized as negligible (*η*^2^ < 0.01), small (*η*^2^ = 0.01–0.06), moderate (*η*^2^ = 0.06–0.14), or large (*η*^2^ > 0.14) [40].

A priori sample size calculations indicated that a total sample size of *n* = 42 patients was needed to detect mean pre-post assessment differences in a three-group setting with moderate effect size (α = 0.05, 1−β = 0.80; f = 0.25). Sample size calculation was performed using the software G*Power v3.1. A moderate effect size was selected to focus on changes that are potentially clinically relevant. However, due to the relatively small sample size in this study, post hoc tests were performed despite the absence of a statistically significant interaction according to the omnibus test in line with previous research [41]. The significance threshold was set at *p* < 0.05. In the case of multiple comparisons, Bonferroni corrections were applied to control for Type-I error inflation. In addition, the analyses were repeated with a comprehensive multivariate analysis approach (3 × 2 design with repeated measures on the second factor). The within-subject factors included two time points (admission: T0, and discharge: T1) at which measurements were taken to capture the temporal effects of the intervention. The multivariate approach allowed for the simultaneous analysis of multiple dependent variables which included the HADS as well as EORTC QLQ-C30 summary scores, ensuring a comprehensive understanding of the intervention’s effects across different dimensions of assessed PROs. This methodological approach allowed us to assess changes over time within subjects while accounting for the correlations among the dependent variables. Effect sizes were reported using partial eta squared (*η*^2^) for both multivariate and univariate analyses. All calculations were performed using IBM SPSS v27.

## 3. Results

### 3.1. HNC Patient Collective

**Figure 1:**
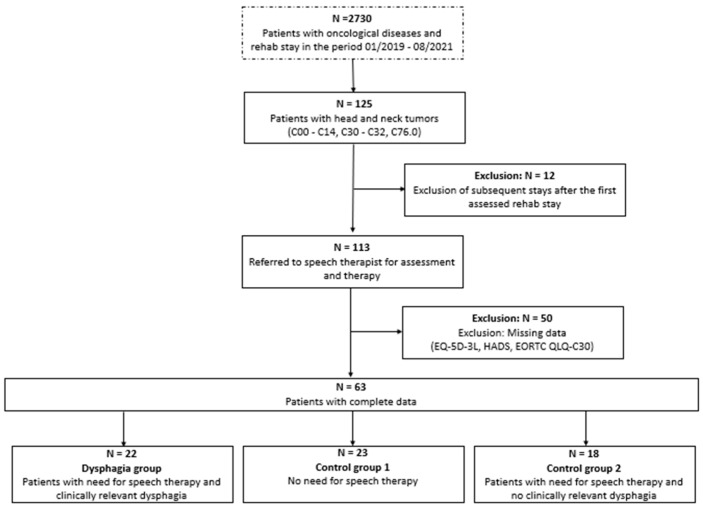
Flow chart data of patient selection. The box with the dotted line shows the overall patient sample in the rehabilitation center, and the boxes with solid lines show the eligible patients with head and neck (HNC) tumors.

Of 2730 patients with various types of cancer, 113 with HNC were identified. Subsequently, 50 patients were excluded according to the prespecified inclusion and exclusion criteria (Figure 1). The remaining 63 participants were stratified into three groups and analyzed.

The cohort consisted predominantly of males (76.2%) with an average age of 60.9 ± 9.2 years (Table 1). Half of the patients (49.2%) had stage IV HNC according to the Union for International Cancer Control (UICC) criteria, and cancer recurrence was evident in 12.7% of these. Notably, no significant differences were identified between the groups regarding gender, age, cancer stage, and recurrence status.

### 3.2. Mean Baseline HRQOL Differences Between the Three Groups

At the initial measurement point (T0), significant between-group differences were observed across multiple functional domains, with patients in the Dysphagia group consistently exhibiting poorer outcomes than those in Control Group 1 and Control Group 2 (Table 2).

A significant group effect was found for global HRQOL (*p* = 0.002, *η*^2^ = 0.184), with post hoc tests indicating that Dysphagia patients had notably lower HRQOL compared to Control Group 1. Similarly, a significant group effect was observed for physical functioning (*p* = 0.030, *η*^2^ = 0.110), where Dysphagia patients demonstrated lower physical functioning than those in Control Group 1.

Social functioning also significantly differed between groups (*p* = 0.001, *η*^2^ = 0.197), with Control Group 1 scoring significantly higher than both the Dysphagia and Control Group 2 patients. Regarding emotional functioning, a significant group effect was found (*p* = 0.024, *η*^2^ = 0.117), with post hoc tests revealing that Dysphagia patients had significantly lower emotional functioning compared to Control Group 1. Furthermore, a significant group effect was observed for the total functional scale (*p* = 0.011, *η*^2^ = 0.141), where post hoc tests confirmed that Dysphagia patients scored significantly lower than Control Group 1. However, no significant group effects were found for role functioning (*p* = 0.068) or cognitive functioning (*p* = 0.101).

With regard to the symptoms, the Dysphagia patients were much more affected by systemic, cancer-related symptoms than patients without a swallowing problem. Their total symptom score was higher than the respective total symptom scores of patients from either control group (Table 3). In detail, their scores for cancer-related fatigue, pain, sleep disturbances, nausea/vomiting, diarrhea, and financial worries were higher than those observed in both control groups. The only symptom that dysphagia patients affected less was dyspnoea. Corresponding to the better functioning scores, patients of Control Group 1 had substantially fewer symptoms than patients who required speech therapy (Control Group 2 and Dysphagia group). A group effect was indicated for fatigue (*p* = 0.071, *η*^2^ = 0.084), although this difference did not achieve statistical significance in post hoc tests (Table 3).

### 3.3. Improvement During Rehabilitation Across Groups

Following rehabilitation, significant time and group effects were observed across multiple functional domains, along with notable group-by-time interactions. As shown in Table 2, global HRQOL significantly improved over time in the Dysphagia group (*p* = 0.002, *η*^2^ = 0.361) and Control Group 2 (*p* = 0.018, *η*^2^ = 0.287). Post hoc tests further indicate that Dysphagia patients and Control Group 2 showed significantly greater improvement over time compared to Control Group 1.

A significant time effect was observed for role functioning in Control Group 2 (*p* < 0.001, *η*^2^ = 0.625), with post hoc tests confirming that Control Group 2 improved more than both the Dysphagia and Control Group 1 patients. Social functioning showed a significant time effect across all groups (*p* < 0.05), with post hoc tests revealing that both the Dysphagia group and Control Group 2 improved more significantly than Control Group 1. Similarly, emotional functioning significantly improved over time across all groups (*p* < 0.05), with post hoc tests confirming that Dysphagia patients experienced significantly greater gains than Control Group 1.

The total functioning scale significantly improved in the Dysphagia group (*p* < 0.001, *η*^2^ = 0.427) and Control Group 2 (*p* < 0.001, *η*^2^ = 0.604). A significant time effect was also observed for physical functioning; however, post hoc tests indicated that the improvements in the Dysphagia group and Control Group 1 differed significantly.

Regarding symptom burden, a statistically significant reduction in fatigue was observed across all groups (*p* < 0.05) (Table 3), with post hoc tests confirming that Dysphagia patients experienced a significantly greater reduction in fatigue than Control Group 1. Sleep disturbances significantly improved in both the Dysphagia group (*p* = 0.006, *η*^2^ = 0.310) and Control Group 2 (*p* = 0.004, *η*^2^ = 0.397). A significant group-by-time interaction was found for sleep disturbances (*p* = 0.034, *η*^2^ = 0.106), with post hoc tests indicating that Dysphagia patients and Control Group 2 improved significantly more than Control Group 1.

Additionally, appetite loss significantly improved over time in both the Dysphagia group (*p* = 0.047, *η*^2^ = 0.175) and Control Group 2 (*p* = 0.035, *η*^2^ = 0.237). The total symptom score also showed statistically significant improvement over time in the Dysphagia group (*p* = 0.041, *η*^2^ = 0.184) and Control Group 2 (*p* = 0.001, *η*^2^ = 0.476).

**Figure 2:**
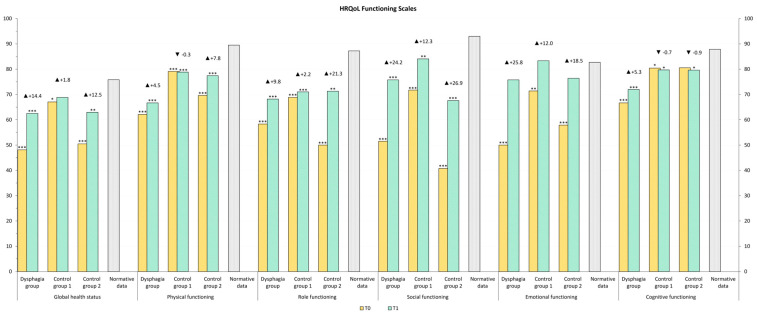
HRQOL, Functioning Scales values after rehabilitation (T1) compared to general population normative data (grey color). The stars represent significant differences between the group and normative data: * *p* ≤ 0.05; ** *p* ≤ 0.01; *** *p* ≤ 0.001.

**Figure 3:**
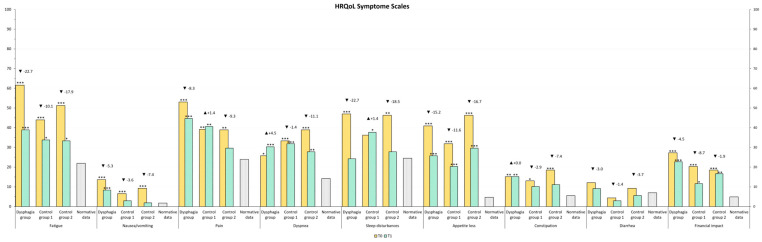
HRQOL Symptom Scales values after rehabilitation (T1) compared to general population normative data (grey color). The stars represent significant differences between the group and normative data: * *p* ≤ 0.05; ** *p* ≤ 0.01; *** *p* ≤ 0.001.

The HRQOL of our study population was compared to normative data from the Austrian population [39]. At T1, emotional functioning in Control Group 1 was comparable to that of the normal population, whereas patients requiring speech therapy exhibited lower scores. Other functional levels remained below those of the normative population. All symptom scores remained above normative levels, except for diarrhea, which was lower in Control Group 1 and Control Group 2 (Figure 2 and Figure 3).

### 3.4. Psychological Distress

Psychological distress was assessed using the HADS questionnaire. Before rehabilitation, anxiety and depression levels were higher in the Dysphagia group and Control Group 2 compared to Control Group 1, though these differences were not statistically significant (Table 4).

At T1, Dysphagia patients showed a statistically significant reduction in anxiety (*p* = 0.015, *η*^2^ = 0.251) and depression (*p* = 0.008, *η*^2^ = 0.291). Control Group 2 also exhibited a significant decrease in depression (*p* = 0.040, *η*^2^ = 0.225) between T0 and T1. In both the Dysphagia group and Control Group 2, these reductions were clinically relevant [42]. Conversely, improvements in anxiety in Control Group 2, as well as reductions in anxiety and depression in Control Group 1, were not statistically significant.

**Table 4:** Anxiety and depression outcomes in cancer rehabilitation: intervention effects.

	Group	T0		T1		Baseline Differences Between Groups		Mean Change During Rehabilitation (Time)		Differences in Mean Changes per Group During Rehabilitation (Time × Group)	
		Mean	SD	Mean	SD	*p*-Value	*η* ^2^	*p*-Value	*η* ^2^	*p*-Value	*η* ^2^
Anxiety [0–21]	Dysphagia group	7.3	4.1	5.1	4.2	0.146	0.062	**0.015**	0.251	0.328	0.037
	Control Group 1	5.0	3.8	4.5	2.6			0.543	0.017		
	Control Group 2	6.3	3.7	4.7	2.7			0.113	0.141		
	Total	6.2	4.0	4.8	3.2			**0.005**	0.122		
Depression [0–21]	Dysphagia group	6.6	4.3	4.5	4.3	0.177	0.056	**0.008**	0.291	0.436	0.027
	Control Group 1	4.6	3.2	3.6	3.3			0.180	0.080		
	Control Group 2	6.4	4.5	4.0	2.4			**0.040**	0.225		
	Total	5.8	4.1	4.0	3.4			**<0.001**	0.197		

All displayed *p*-values are Bonferroni corrected to control for Type-I error inflation. Bold text—*p* < 0.05; ^a^ *p* < 0.05 between dysphagia group and Control Group 1; ^b^ *p* < 0.05 between dysphagia group and control group 2; ^c^ *p* < 0.05 between Control Group 1 and Control Group 2. Effect size values *η*^2^ ≥ 0.01 were considered small, *η*^2^ ≥ 0.06 as medium, and *η*^2^ ≥ 0.14 as large. Mean differences between T0 and T1 surpassing clinically relevant cut-off levels are highlighted in green: 1.3 points for anxiety and 1.4 points for depression [43]. Group size: Dysphagia group—22 patients, Control Group 1–23 patients, Control Group 2–18 patients.

**Figure 4:**
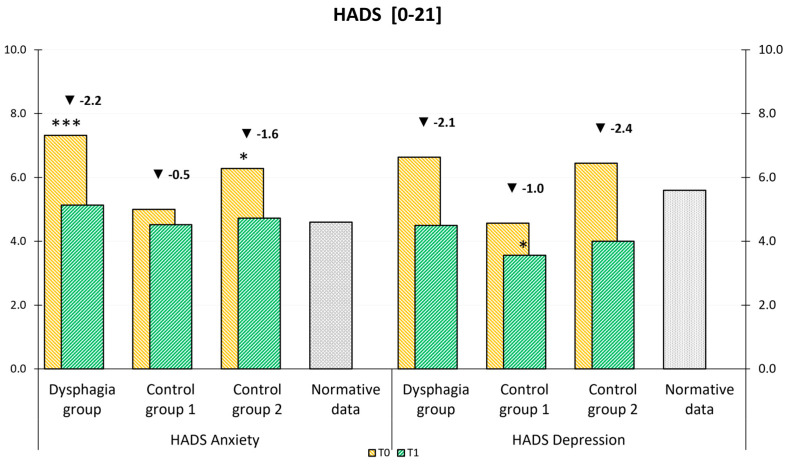
HADS mean values at admission (T0) and discharge (T1) compared to general population normative data (a–b). The stars represent significant differences between the group and normative data: * *p* ≤ 0.05; ** *p* ≤ 0.01; *** *p* ≤ 0.001.

As shown in Figure 4, a comparison with normative data from the Central European population revealed that anxiety levels at T1 in our study groups were comparable to those in the general population [32]. Notably, depression levels in Control Group 1 were even lower than those observed in the normal population. An analysis of group-by-time interactions showed no statistically significant differences in improvement between the three groups.

### 3.5. Multivariate Analysis

A comprehensive multivariate analysis was conducted to examine the impact of the intervention on different groups over time. Our results showed a pronounced temporal effect across all outcome measures. In addition, the multivariate analysis highlighted the holistic impact of the interventions on multiple outcomes with a high effect size (*η*^2^ = 0.421). Distinct group effects emerged, particularly in the improvement of the QLQ-C30 Global Health Status (*η*^2^ = 0.124), and were further highlighted in the multivariate analysis (*η*^2^ = 0.123). The intervention had multiple effects, most notably improving functional aspects, reducing psychological distress, and promoting overall well-being in HNC patients (see Table 5).

## 4. Discussion

### 4.1. Unmet Needs: Impact of Dysphagia and Speech Disorders on HRQOL and Functioning

This study examined the effects of oncological rehabilitation on the HRQOL of HNC survivors using routinely collected patient-reported outcomes (PROs). The questionnaires completed before rehabilitation helped identify patients’ unmet needs. Dysphagia patients demonstrated significantly poorer HRQOL compared to other HNC patients and the general Austrian population. This was reflected in a lower total functioning score, primarily due to reduced physical, emotional, and cognitive functioning. Additionally, Dysphagia patients reported a higher total symptom score, characterized by increased fatigue, nausea/vomiting, pain, diarrhea, and financial concerns.

Late adverse effects are reported in more than half of HNC survivors and include fatigue, joint pain, muscle aches, sleep disturbances, sensitivity to cold, and neurocognitive symptoms [42]. Fatigue, in particular, may persist long-term, especially after radiotherapy [44]. Our findings align with previous reports indicating that speech and swallowing disorders are associated with impaired HRQOL [28,42]. Dysphagia has also been linked to adverse effects such as coughing, sleep disturbances, and reduced mouth opening [45], further increasing the risk of dehydration, malnutrition, aspiration, and pneumonia [3,46,47]. The results of this study highlight that dysphagia has far-reaching systemic consequences that extend beyond swallowing function, impacting multiple aspects of physical, mental, and social health.

Initially, we planned to compare dysphagia patients with those without impaired swallowing. However, the control group was found to be heterogeneous, necessitating an alternative approach. Control Group 2 exhibited significantly lower HRQOL, as well as reduced emotional and social functioning, compared to Control Group 1. These patients were also more affected by fatigue and gastrointestinal symptoms. Both Dysphagia and Control Group 2 patients experienced more severe symptoms (except pain) than Control Group 1 and the general population [32].

Dysphagia patients also exhibited higher levels of anxiety and depression than both control groups. These findings align with previous research showing that 23% of HNC patients experience significant depression [48]. A recent study from the U.S. reported persistent psychological distress among HNC survivors, with 15.1% of those treated with irradiation experiencing depression and 10.0% reporting anxiety after 24 months [49]. Our previous study comparing various cancer types also found elevated depression levels in HNC patients, although their anxiety levels were comparable to the broader cancer survivor population [28]. We hypothesize that the heightened fear of aspiration and pneumonia may be a major contributor to this anxiety.

### 4.2. Effects of Cancer Rehabilitation

The effectiveness of rehabilitation was assessed by comparing PROs at admission and discharge. Group-by-time effect analyses were conducted to identify between-group differences in rehabilitation outcomes. All three HNC subgroups demonstrated significant improvements in global HRQOL, as well as in total functional and total symptom scores, with the most pronounced benefits observed in Dysphagia patients and Control Group 2.

Social and emotional functioning improved across all groups, with large effect sizes, while reductions in fatigue, insomnia, and appetite loss contributed to the overall increase in HRQOL. Consistent with previous findings, we and others have reported that rehabilitation can enhance HRQOL, as well as mental, social, and physical functioning in cancer survivors across various cancer types [28,50]. Furthermore, a randomized trial examining swallowing interventions combined with resistance training during radiotherapy reported improvements in mouth opening, depression, anxiety, pain, and insomnia, as well as a reduction in the use of pain medication. However, it is noteworthy that swallowing function did not improve significantly more than in the control group [51]. Another randomized trial found improvements in HRQOL and all functional domains of the EORTC QLQ-C30 following nursing interventions. These interventions included guidance on mouth-opening exercises, neck massage, oral coordination training, and direct feeding training for patients with esophageal cancer undergoing radiotherapy [35]. The results presented here suggest that, among all HNC patients, those with the greatest need for rehabilitation—particularly dysphagia patients—experience the most substantial benefits. Speech therapy is highly relevant for HNC patients who suffer from dysphagia or other symptoms of oral dysphagia patients’ function. For those patients who are not primarily affected by dysphagia (in our study: Control Group 2), it has been shown in a randomized–controlled trial that voice rehabilitation exerts positive effects on voice function and HRQOL, which persist up to 12 months of follow-up and appear to prevent deterioration of perceived roughness [52].

Evidence-based recommendations for the rehabilitation of dysphagia patients undergoing radiotherapy have recently been published in a systematic review, recommending early identification by dysphagia screening, highlighting physiotherapy and prophylactic swallowing training, and suggesting feeding management, pain control, and oral care [53]. Methods of functional dysphagia therapy and manual swallowing therapy were employed to improve the range of motion and to strengthen muscles relevant to swallowing, which includes strength and range of motion for lips, tongue, and larynx [3,54]. Additionally, compensatory maneuvers such as the super-supraglottic swallow and the Mendelsohn maneuver were developed with the patients, adapted to altered structural conditions following surgery and/or radiotherapy, as well as posture, movement, and relaxation exercises, and counseling, e.g., for dryness of mouth, and adaptive measures were carried out. At least small to moderate effectiveness of swallowing exercises has been demonstrated, especially in improving the function of swallowing, and mouth opening in HNC patients undergoing multimodal treatment [55].

The specific effects of speech therapy in the context of rehabilitation of HNC patients remain controversial. A randomized–controlled trial, that investigated the effects of head-lift exercises in HNC patients with dysphagia according to instruction by a speech and language pathologist, revealed improvement of role function after 8 weeks, and of HRQOL and social function after 12 months, but no significant differences between groups [56]. The authors concluded that no convincing results of improvement have been found in patients’ perception of general and dysphagia-specific HRQOL. Moreover, in contrast to our study, a meta-analysis on the effects of exercise-based rehabilitation in HNC patients, described no differences in quality of life at any of the follow-up periods [57]. In the latter study, exercise was minimally superior to controls in reducing fatigue in HNC patients who were treated with chemo-radiotherapy. A recently published randomized trial investigating prophylactic swallowing exercises in patients with laryngectomy, however, has revealed the lasting efficacy of swallowing training in laryngectomized patients [58]. In addition, it was confirmed in a recently published systematic review that home-based approaches utilizing telerehabilitation also improve the safety of swallowing and oral feeding, nutritional status, and swallowing-related quality of life, and improve negative emotions [34].

Dysphagia patients experienced a significant improvement in appetite loss in our study. These patients are severely affected by their inability to eat normally. Eating and food have important biological and social functions in people’s lives [59]. In addition to dysphagia, conditions like dysgeusia, oral mucositis, thickened saliva, sore throat, xerostomia, and pain or high sensitivity while eating spicy food impede their quality of life. This may impact social contacts and, as a result, some patients tend to eat alone [59]. It is therefore conceivable that effective amelioration of dysphagia by speech therapists may result in improved social function. In addition, improvement of appetite as noted in our study can potentially help to stabilize food intake and counteract further weight loss. It has been demonstrated that swallowing training combined with nutritional intervention can improve not only the swallowing function but also the nutritional status and the quality of life of laryngeal cancer patients with dysphagia after operation and radiotherapy [60]. Thus, the observed improvement of systemic symptoms and social functioning in our study is likely to result from the interplay of therapeutic modalities. Physiotherapy, medical training therapy, occupational therapy, dietary advice, and psychological counseling contribute to the effectiveness of rehabilitation [61,62]. The effectiveness of exercise interventions has been shown for HNC patients receiving chemoradiotherapy [12]. In this randomized–controlled trial, improvement in the functional capacity, quality of life, and prevention of worsening of fatigue were observed in the exercise group but not in the control. Hence, structured and multidisciplinary rehabilitation programs may potentially be effective in multiple dimensions whereas a guided self-help exercise program for laryngectomized patients improved swallowing, but failed to improve speech, shoulder problems, and quality of life [63].

Swallowing ability and depression have been identified as the most important factors associated with dysphagia-specific HRQOL [33]. In our study, we observed notable improvements in anxiety and depression with a large effect in the groups of Dysphagia and Control Group 2 patients. In fact, in Dysphagia patients, and in both control groups, depression was reduced even below the level described in the general population [31]. Hence, while the negative impact of dysphagia on emotional and mental health is well-known from recent studies [64,65], our study shows that it can be effectively alleviated by psychosocial counseling as an integrative component of a three-week rehabilitation program.

It is, however, noteworthy that, despite the improvements during the rehabilitation measure, with the exception of the emotional functioning of Control Group 1 patients, all functioning scores remained below those reported by the general population at T1. This underlines the generally high physical and psychosocial burden on HNC patients, which can only be partially overcome by rehabilitation. Our results may, however, reflect a high number of patients with advanced-stage HNC with 73.0% being diagnosed with UICC stage III–IV. In contrast, quality of life scores returned to baseline values in 70.3% of patients with early-stage HNC (UICC I–II) after 12 months in a study by Ihara et al. [66]. In their study, normalization of oral and swallowing functions did not predict the return to baseline values for quality of life. The authors emphasized the need to treat these patients with consideration of several factors such as the patient’s social and psychological condition.

We addressed the specific needs of HNC survivors, emphasizing that a direct comparison among dysphagia patients, HNC survivors requiring speech therapy for other reasons, and those without local impairments has not been previously reported. Dysphagia patients experience more severe fatigue, pain, sleep disturbances, gastrointestinal issues, and financial concerns, along with significantly reduced physical, social, and emotional functioning and overall HRQOL. Additionally, HNC survivors requiring speech therapy without dysphagia (Control Group 2) show lower HRQOL and functioning compared to unaffected individuals (Control Group 1), with notably poor social functioning. A three-week multimodal rehabilitation program significantly improves symptoms and functioning across all HNC survivor groups, with the most pronounced benefits seen in dysphagia patients.

### 4.3. Strengths and Limitations

A key strength of this study is the direct comparison of different subgroups of HNC survivors, allowing for meaningful conclusions about each group’s burden and response to therapeutic interventions. Our cohort of previously unselected cancer survivors undergoing rehabilitation provides valuable real-world data on unmet needs. To control for Type I error inflation (i.e., false positive results in multiple hypothesis testing), we applied Bonferroni-corrected *p*-values and conducted a multivariate analysis, which supported our initial findings.

However, the study also has several limitations. One potential limitation is the sample size of 63 patients, which may have influenced the observed effect sizes. While a priori power calculations indicated that the study was adequately powered, additional subgroup analyses—such as the impact of UICC stage, severity of dysphagia, and type of oncological therapy—were not feasible. The number of evaluable patients was lower than expected due to restricted admission to the rehabilitation center, including its temporary closure during the COVID-19 pandemic. Additionally, as data collection was part of routine clinical procedures, not all patients completed the questionnaires by the end of their rehabilitation stay.

Furthermore, while this single-center analysis ensured standardized patient assessments and therapies, the findings may not be fully generalizable to other centers or countries. Another limitation is that most patients had an ECOG status of 0–2, as the Austrian pension fund does not refer patients with very poor general health to rehabilitation centers. Lastly, although the QLQ-C30 was used as part of standard rehabilitation procedures, no dysphagia-specific questionnaires or objective assessments—such as fiberoptic endoscopic evaluation of swallowing (FEES) or structured dysphagia therapy programs—were utilized [59]. This was not feasible in the context of a general cancer rehabilitation program that was not exclusively designed for HNC patients. Nonetheless, our study highlights the significant benefits of general oncological rehabilitation for these patients.

### 4.4. Considerations for Further Research

Since the data for our study was collected from clinical routine procedures in a rehabilitation center where all cancer entities were treated, no PROs on the specific conditions of HNC patients were utilized. Further research should employ instruments like the EORTC H&N43 together with the QLQ-C30 questionnaire [67]. This instrument contains questions that analyze topics such as swallowing disorders in detail, e.g., problems swallowing liquids, pureed food, solid food, whether there were choking at-tacks when swallowing, or whether there were problems with the teeth. Similarly, the MD Anderson symptom inventory for head and neck cancer (MDASI-HN) is a disease-specific module containing questions specific to HNC along with questions on core symptoms and interference [68], and the FACT-HN analyses additional concerns with respect to HNC together with physical, social/family, emotional and functional well-being [69]. In addition to PROs, clinician-reported outcome measurements are useful for an objective description of dysphagia-associated malfunction, e.g., the cancer-specific swallowing assessment tool Mann Assessment of Swallowing Ability-Cancer MASA-C [70]. Moreover, instrumental assessments like the above-mentioned FEES and videofluoroscopy are considered standard methods for dysphagia screening [71]. Studies specifically designed for the analysis of dysphagia-associated impairments should incorporate such test systems for optimal description of the local symptoms and disabilities.

## 5. Conclusions

Our results indicate that dysphagia—and, to a lesser extent, other speech-related disabilities—leads to poorer quality of life in HNC patients. This is due to reduced functioning across multiple domains, particularly in physical, emotional, and social aspects. Symptomatically, these patients are more affected by cancer-related fatigue, sleep disturbances, and appetite loss. In conclusion, impairments in swallowing or speech are associated not only with localized dysfunction but also with widespread systemic impairments, which are even more pronounced in patients with dysphagia.

The observed improvements in emotional and social functioning are likely the result of a multidisciplinary, multimodal rehabilitation approach involving physiotherapists, occupational therapists, dietitians, and psychologists. Although the cancer rehabilitation program was not specifically designed to address the needs of dysphagia patients, our findings suggest that these highly disabled and psychologically distressed individuals still benefit significantly. In fact, the extent of improvement may be even greater in dysphagia patients than in other HNC survivors.

Our study emphasizes that HNC patients with dysphagia have specific needs that go far beyond the purely local problems of swallowing. In addition to speech therapy and ensuring adequate nutrition, it is also important to consider the pronounced impairments in emotional, social, and role function. For the care of these patients in clinical practice, it is important to know that fatigue, sleep disorders, and inappetence as well as anxiety and depression can be treated effectively, which can significantly improve their quality of life.

## Figures and Tables

**Table 1:** Patient characteristics.

	Total Sample	Dysphagia Patients	Control Group 1	Control Group 2	*p*-Value
	N (%)	N (%)	N (%)	N (%)	
**Sex**					
Male	48 (76.2)	15 (68.2)	20 (87.0)	13 (72.2)	0.301
Female	15 (23.8)	7 (31.8)	3 (13.0)	5 (27.8)	
**Age**					
Mean (SD)	60.9 (9.2)	59.8 (7.1)	61.1 (9.5)	62.1 (11.2)	0.725
**UICC**					
I	3 (4.8)	0	3 (13.0)	0	0.19
II	7 (11.1)	2 (9.1)	3 (13.0)	2 (11.1)	
III	15 (23.8)	4 (18.2)	7 (30.4)	4 (22.2)	
IV	31 (49.2)	13 (59.1)	7 (30.4)	11 (61.1)	
Missing	7 (11.1)	3 (13.6)	3 (13.0)	1 (5.6)	
**Recurrent Disease**					
Yes	8 (12.7)	3 (13.6)	3 (13.0)	2 (11.1)	0.97
No	55 (87.3)	19 (86.4)	20 (87.0)	16 (88.9)	

N—sample Size; UICC (I, II, III, IV)—cancer staging system; *p*-value—the difference between groups.

**Table 2:** Health-related quality of life outcomes in cancer rehabilitation: intervention effects on functioning scales.

	Group	T0		T1		Baseline (T0) Differences Between Groups		Mean Changes (T1–T0) During Rehabilitation (Time)		Differences in Mean Changes per Group During Rehabilitation (Time × Group)	
		Mean	SD	Mean	SD	*p*-Value	*η* ^2^	*p*-Value	*η* ^2^	*p*-Value	*η* ^2^
Global health/QOL [0–100]	Dysphagia group	48.1	17.2	62.5	20.4	**0.002** ^a,c^	0.184	**0.002**	0.361	0.076 ^a,c^	0.082
	Control Group 1	67.0	19.4	68.8	15.9			0.648	0.010		
	Control Group 2	50.5	19.5	63.0	18.8			**0.018**	0.287		
	Total	55.7	20.3	64.9	18.3			**<0.001**	0.200		
Physical functioning [0–100]	Dysphagia group	62.1	23.1	66.7	24.4	**0.030** ^a^	0.110	0.252	0.062	0.317 ^a^	0.038
	Control Group 1	79.1	19.6	78.8	22.3			0.927	0.000		
	Control Group 2	69.6	20.0	77.4	21.1			0.087	0.162		
	Total	70.5	21.9	74.2	23.0			0.068	0.054		
Role functioning [0–100]	Dysphagia group	58.3	29.4	68.2	29.1	0.068	0.086	0.067	0.151	**0.027** ^c^	0.114
	Control Group 1	68.8	24.3	71.0	26.7			0.657	0.009		
	Control Group 2	50.0	21.4	71.3	21.2			**<0.001**	0.625		
	Total	59.8	26.2	70.1	25.8			**<0.001**	0.211		
Social functioning [0–100]	Dysphagia group	51.5	30.4	75.8	29.9	**0.001** ^a,c^	0.197	**0.004**	0.337	0.204 ^a,c^	0.052
	Control Group 1	71.7	24.8	84.1	17.0			**0.035**	0.188		
	Control Group 2	40.7	23.0	67.6	28.9			**<0.001**	0.658		
	Total	55.8	29.0	76.5	26.0			**<0.001**	0.371		
Emotional functioning [0–100]	Dysphagia group	50.0	29.7	75.8	28.2	**0.024** ^a^	0.117	**<0.001**	0.525	0.135 ^a^	0.065
	Control Group 1	71.4	23.4	83.3	16.3			**0.007**	0.284		
	Control Group 2	57.9	23.1	76.4	17.9			**0.004**	0.393		
	Total	60.1	26.9	78.7	21.5			**<0.001**	0.414		
Cognitive functioning [0–100]	Dysphagia group	66.7	30.9	72.0	31.9	0.101	0.074	0.390	0.035	0.601	0.017
	Control Group 1	80.4	17.9	79.7	21.9			0.840	0.002		
	Control Group 2	80.6	20.8	79.6	25.9			0.859	0.002		
	Total	75.7	24.5	77.0	26.7			0.675	0.003		
Total functioning scale score [0–100]	Dysphagia group	57.7	23.7	71.7	23.5	**0.011** ^a,c^	0.141	**<0.001**	0.427	0.054 ^a,c^	0.093
	Control Group 1	74.3	17.1	79.4	16.6			0.080	0.133		
	Control Group 2	59.8	15.1	74.5	16.7			**<0.001**	0.604		
	Total	64.4	20.4	75.3	19.3			**<0.001**	0.393		

All displayed *p*-values are Bonferroni corrected to control for Type-I error inflation. Bold text—*p* < 0.05; ^a^ *p* < 0.05 between dysphagia group and Control Group 1; ^b^ *p* < 0.05 between dysphagia group and control group 2; ^c^ *p* < 0.05 between Control Group 1 and Control Group 2. Effect size values *η*^2^ ≥ 0.01 were considered small, *η*^2^ ≥ 0.06 as medium, and *η*^2^ ≥ 0.14 as large. Group size: Dysphagia group—22 patients, Control Group 1–23 patients, Control Group 2–18 patients. Details of post hoc tests are described in Section 2.6. Post hoc group comparisons.

**Table 3:** Health-related quality of life outcomes in cancer rehabilitation: intervention effects on symptom scales.

	Group	T0		T1		Baseline (T0) Differences Between Groups		Mean Changes During Rehabilitation (Time)		Differences in Mean Changes per Group During Rehabilitation (Time × Group)	
		Mean	SD	Mean	SD	*p*-Value	*η* ^2^	*p*-Value	*η* ^2^	*p*-value	*η* ^2^
Fatigue [0–100]	Dysphagia group	61.6	26.3	38.9	26.1	0.071	0.084	**0.002**	0.370	0.222 ^a^	0.049
	Control Group 1	44.0	28.9	33.8	22.8			**0.013**	0.249		
	Control Group 2	51.2	17.9	33.3	20.5			**0.004**	0.399		
	Total	52.2	26.0	35.4	23.2			**<0.001**	0.336		
Nausea/ vomiting [0–100]	Dysphagia group	13.6	16.0	8.3	17.6	0.290	0.040	0.296	0.052	0.813	0.007
	Control Group 1	6.5	9.7	2.9	9.6			0.203	0.072		
	Control Group 2	9.3	19.2	1.9	5.4			0.104	0.148		
	Total	9.8	15.2	4.5	12.4			**0.025**	0.081		
Pain **(0–100)**	Dysphagia group	53.0	28.0	44.7	31.9	0.160	0.059	0.185	0.082	0.314	0.038
	Control Group 1	39.1	27.3	40.6	30.9			0.780	0.004		
	Control Group 2	38.9	26.2	29.6	23.3			0.096	0.154		
	Total	43.9	27.6	38.9	29.5			0.100	0.044		
Dysphagia patient dyspnoea **(0–100)**	Dysphagia group	25.8	32.4	30.3	32.4	0.450	0.026	0.378	0.037	0.149	0.062
	Control Group 1	33.3	37.6	31.9	32.5			0.803	0.003		
	Control Group 2	38.9	26.2	27.8	28.6			0.055	0.200		
	Total	32.3	32.8	30.2	30.9			0.401	0.012		
Sleep disturbances [0–100]	Dysphagia group	47.0	33.6	24.2	29.4	0.477	0.024	**0.006**	0.310	**0.034** ^a,c^	0.106
	Control Group 1	36.2	30.0	37.7	33.8			0.847	0.002		
	Control Group 2	46.3	34.6	27.8	30.8			**0.004**	0.397		
	Total	42.9	32.5	30.2	31.5			**0.002**	0.149		
Appetite loss [0–100]	Dysphagia group	40.9	34.0	25.8	29.0	0.438	0.027	**0.047**	0.175	0.866	0.005
	Control Group 1	31.9	35.5	20.3	32.9			0.073	0.139		
	Control Group 2	46.3	39.8	29.6	37.7			**0.035**	0.237		
	Total	39.2	36.2	24.9	32.8			**<0.001**	0.181		
Constipation **(0–100)**	Dysphagia group	15.2	24.6	15.2	24.6	0.783	0.008	1.000	0.000	0.557	0.019
	Control Group 1	13.0	19.4	10.1	23.4			0.492	0.022		
	Control Group 2	18.5	30.7	11.1	19.8			0.104	0.148		
	Total	15.3	24.6	12.2	22.6			0.212	0.026		
Diarrhea **(0–100)**	Dysphagia group	12.1	24.2	9.1	23.4	0.507	0.022	0.680	0.008	0.940	0.002
	Control Group 1	4.3	20.9	2.9	13.9			0.328	0.043		
	Control Group 2	9.3	22.3	5.6	17.1			0.163	0.111		
	Total	8.5	22.4	5.8	18.5			0.317	0.017		
Financial impact **(0–100)**	Dysphagia group	27.3	31.9	22.7	29.8	0.560	0.019	0.378	0.037	0.662	0.014
	Control Group 1	20.3	26.1	11.6	19.1			0.056	0.157		
	Control Group 2	18.5	23.5	16.7	23.6			0.790	0.004		
	Total	22.2	27.4	16.9	24.6			0.108	0.042		
Total symptom scales score **(0–100)**	Dysphagia group	32.9	18.4	24.4	20.1	0.318	0.038	**0.041**	0.184	0.353	0.034
	Control Group 1	25.4	17.1	21.3	13.9			0.120	0.106		
	Control Group 2	30.8	14.9	20.4	14.3			**0.001**	0.476		
	Total	29.6	17.0	22.1	16.3			**<0.001**	0.227		

All displayed *p*-values are Bonferroni corrected to control for Type-I error inflation. Bold text—*p* < 0.05; ^a^ *p* < 0.05 between dysphagia group and Control Group 1; ^b^ *p* < 0.05 between dysphagia group and control group 2; ^c^ *p* < 0.05 between Control Group 1 and Control Group 2. Effect size values *η*^2^ ≥ 0.01 were considered small, *η*^2^ ≥ 0.06 as medium, and *η*^2^ ≥ 0.14 as large. Group size: Dysphagia group—22 patients, Control Group 1–23 patients, Control Group 2–18 patients.

**Table 5:** Analysis of intervention effects (*η*^2^) on the outcomes between and within the subjects.

	Mean Change During Rehabilitation (Time, η2)	Differences Between Groups (η2)	Mean Change During Rehabilitation per Group (Time × Group, η2)
HADS Anxiety	0.122 **	0.041	0.037
HADS Depression	0.197 ***	0.042	0.027
QLQ Functioning scales	0.393 ***	0.088	0.093
QLQ Symptom scales	0.227 ***	0.023	0.034
QLQ Global health status	0.200 ***	0.124 *	0.082
Multivariate	0.421 ***	0.123	0.075

* *p* ≤ 0.05; ** *p* ≤ 0.01; *** *p* ≤ 0.001. Group size: Total—63, Dysphagia group—22 patients, Control Group 1–23 patients, Control Group 2–18 patients.

## Data Availability

The research data supporting this publication are stored at our institutional digital data repository for published research accessible via https://creed.lbg.ac.at (accessed on 28 December 2024). The data sets analyzed in this manuscript are not publicly available due to ethical and legal restrictions (data contain potentially identifying and sensitive patient information). However, pseudonymized data sets have been created for the purpose of re-use and are also accessible via creed.lbg.ac.at. Requests for access to anonymized data sets should be directed to the corresponding authors.
